# Inter-individual variation in health and disease associated with pulmonary infectious agents

**DOI:** 10.1007/s00335-018-9733-z

**Published:** 2018-01-20

**Authors:** Kirsten C. Verhein, Heather L. Vellers, Steven R. Kleeberger

**Affiliations:** 1 Inflammation, Immunity, and Disease Laboratory, National Institute of Environmental Health Sciences, National Institutes of Health, Research Triangle Park, NC USA; 2 Inflammation, Immunity, and Disease Laboratory, National Institute of Environmental Health Sciences, National Institutes of Health, 111 T.W. Alexander Dr., Building 101, Rm. D240, Research Triangle Park, NC 27709 USA

## Abstract

Respiratory infectious diseases resulting from bacterial or viral pathogens such as *Mycobacterium tuberculosis, Streptococcus pneumoniae*, respiratory syncytial virus (RSV), or influenza, are major global public health concerns. Lower respiratory tract infections are leading causes of morbidity and mortality, only behind ischemic heart disease and stroke (GBD 2015 LRI Collaborators in Lancet Infect Dis 17(11):1133–1161, 2017). Developing countries are particularly impacted by these diseases. However, while many are infected with viruses such as RSV (> 90% of all individuals are infected by age 2), only sub-populations develop severe disease. Many factors may contribute to the inter-individual variation in response to respiratory infections, including gender, age, socioeconomic status, nutrition, and genetic background. Association studies with functional single nucleotide polymorphisms in biologically plausible gene candidates have been performed in human populations to provide insight to the molecular genetic contribution to pulmonary infections and disease severity. In vitro cell models and genome-wide association studies in animal models of genetic susceptibility to respiratory infections have also identified novel candidate susceptibility genes, some of which have also been found to contribute to disease susceptibility in human populations. Genetic background may also contribute to differential efficacy of vaccines against respiratory infections. Development of new genetic mouse models such as the collaborative cross and diversity outbred mice should provide additional insight to the mechanisms of genetic susceptibility to respiratory infections. Continued investigation of susceptibility factors should provide insight to novel strategies to prevent and treat disease that contributes to global morbidity and mortality attributed to respiratory infections.

## Introduction

The World Health Organization (WHO) has estimated that two of the top ten causes of global mortality are due to infectious diseases of the lung. Lower respiratory infections, including influenza, pneumonia, *Haemophilus influenzae* type B (Hib), and respiratory syncytial virus (RSV), account for over 3 million deaths per year (approximately 43/100,000); tuberculosis causes over 1.8 million deaths per year (approximately 19/100,000). Though most will have a respiratory infection in their lifetime, there is significant inter-individual variation in susceptibility to severity of infection and disease progression. These differences have been attributed to a number of extrinsic (environmental) and intrinsic (host) factors. Extrinsic factors that could contribute to inter-individual variation include socioeconomic status, exposure to environmental stimuli (e.g., air pollution), nutrition, and co-exposures/infections. Intrinsic factors include age, sex, pre-existing disease, and genetic background. Alone or combined, these factors have been shown to influence individual pulmonary responses to infectious and non-infectious agents, as well as vaccines against infections.

**Fig. 1:**
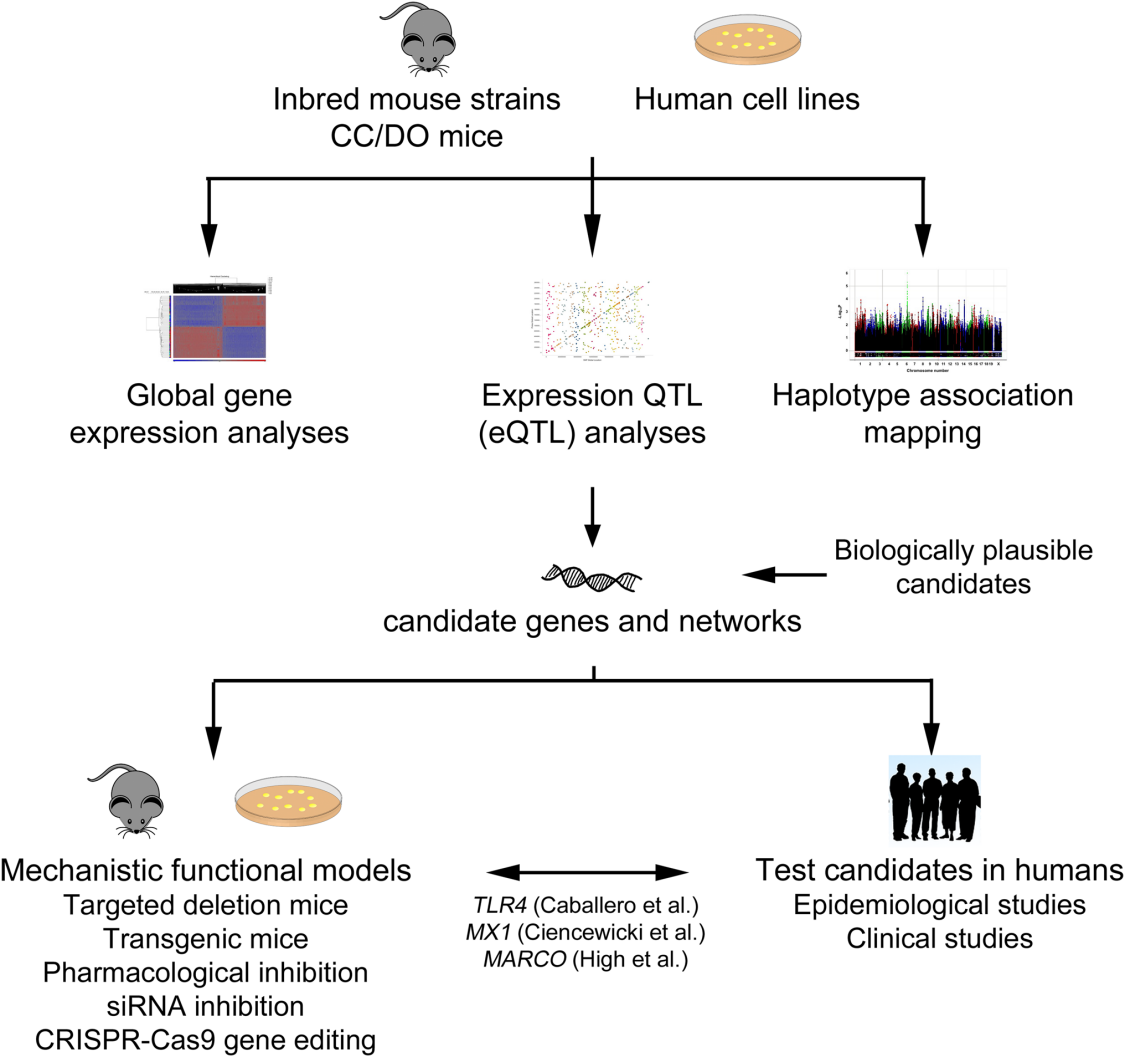
A schematic representing potential steps and models for candidate susceptibility gene discovery. Initial studies could utilize mouse models, including inbred strains, collaborative cross (CC), and/or diversity outbred (DO) mice, or panels of human cell lines, such as lymphoblastoid cells, for gene expression analyses, eQTL, or haplotype association mapping. Candidate genes from these approaches, along with other biologically plausible genes, can be tested for mechanism using additional methods in mice or cells and also in human populations. Examples of genes where these approaches have been used successfully include *TLR4* (Toll-like receptor 4), *MX1* (myxovirus (influenza virus) resistance 1), and *MARCO* (macrophage receptor with collagenous structure)

This review will focus on the role of genetic background on susceptibility to respiratory infection as well as subsequent disease progression. We discuss human population studies that have tested the roles of functional single nucleotide polymorphisms (SNPs) in biologically plausible gene candidates in respiratory infectious disease to determine potential susceptibility mechanisms. We also review genome-wide association studies (GWAS) that have been performed in inbred mouse models of respiratory infections. These models have provided insight to novel susceptibility genes and mechanisms that can be tested in human populations (Fig. 1). Finally, we discuss future directions that may be pursued to better understand the genetic contribution to inter-individual variation in respiratory infectious disease and strategies to prevent disease.

## Host genetic susceptibility to bacterial infection

### *Mycobacterium tuberculosis*

*Mycobacterium tuberculosis* is estimated to infect one-third of the world’s population and globally remains a major cause of morbidity and mortality (World Health Organization 2013). Only about 10% of infected individuals develop tuberculosis disease (TB) (Sudre et al. 1992) and host genetic background contributes to TB susceptibility. Based on tuberculin skin tests, those of European descent have a lower risk of infection than those of African descent [summarized in Stead et al. (1990)], and monozygotic twins have a 2.5-fold higher concordance for TB than dizygotic twins (Comstock 1978). Increased susceptibility to TB infection is also seen in those with the rare inherited disorder Mendelian susceptibility to mycobacterial disease (MSMD). These individuals are more vulnerable to weakly virulent mycobacteria and they carry mutations interferon-gamma/interleukin 12 pathway genes [reviewed in Bustamante et al. (2014)]. The role of host genetic background in TB susceptibility has recently been reviewed (Kinnear et al. 2017), therefore only highlights will be provided here.

A number of candidate gene association studies have been conducted that focus on SNPs in immunity genes, the first of which was solute carrier family 11 member 1 *SLC11A1* (formerly referred to as *NRAMP1*) (Bellamy et al. 1998). *SLC11A1* encodes a divalent cation transporter on phagosomal membranes and the association of four polymorphisms in the gene have subsequently been inconsistently associated with TB susceptibility. Despite the inconsistencies among studies, meta-analyses have found associations between polymorphisms in *SLC11A1* and TB susceptibility in Asian and African populations (Meilang et al. 2012). In a Chinese Han population, a polymorphism in the scavenger receptor *MARCO* (macrophage receptor with collagenous structure) was found to be associated with increased risk of tuberculosis (Lao et al. 2017). Another candidate immunity gene, toll-like receptor 8 (*TLR8*), has also been associated with susceptibility to TB, particularly in males compared to females since *TLR8* is located on the X chromosome (Davila et al. 2008; Salie et al. 2015). Variants found in X chromosome genes may at least partially explain why males are more affected by TB than females. Several additional genes have also had inconsistent associations with TB susceptibility across studies, including the vitamin D receptor (*VDR*) and the human leukocyte antigen DRB1 (*HLA-DRB1*). As demonstrated above, meta-analyses are often used to address inconsistencies among studies. For example, meta-analyses demonstrated an association of a polymorphism in *VDR* in HIV-negative TB patients (Huang et al. 2015; Xu et al. 2015). However, even results from meta-analyses can be mixed, particularly for *HLA-DRB1*, with some alleles conferring protection and others susceptibility depending on the population studied (Tong et al. 2015; Yang et al. 2016).

As with humans, inbred mice are differentially susceptible to *Mycobacterium tuberculosis* infection (Medina and North 1998; Orme 2003) and have been used to discover candidate TB susceptibility genes including HLA-H2 and *Ipr1* (Kramnik et al. 2000; Logunova et al. 2015; Pan et al. 2005). The contribution of mouse models to candidate gene discovery has recently been reviewed (Apt et al. 2017; Kramnik and Beamer 2016).

Human GWAS have been performed to search for variants across the genome that associate with disease and have each identified different significant regions that appear to depend on the population used for the study [summarized in Kinnear et al. (2017)]. To address inconsistencies among studies, a consortium was developed to combine all TB GWAS datasets into a database which will be used for meta-analyses. The consortium will benefit from a large sample size that is inclusive of multiple ethnicities (Naranbhai 2016).

Further complicating the identification of host susceptibility to TB is the interaction between host and bacterial genomes. Variants in human candidate genes have been associated with bacterial genotypes in specific populations (Caws et al. 2008; Salie et al. 2014; Thye et al. 2011). For example, a SNP in Toll-like receptor 2 (*TLR2*) was found in individuals in Vietnam who were more likely to be infected with Lineage 2 strains from the Beijing family (Caws et al. 2008). Recent work has utilized GWAS data to look for associations with bacterial lineage. After stratifying human GWAS with bacterial lineage, Omae et al. (2017) found a SNP near the CD35 gene that was associated with old age onset cases of TB, suggesting host genetic risk may also depend on the genetic makeup of the pathogen. Future efforts that include combining GWAS studies and including bacterial lineage should prove useful to examine the role of genetic background in various human infectious diseases.

Host genetics also likely plays a role in tuberculosis vaccine efficacy. There is a single vaccine available for TB, Bacillus Calmette–Guérin (BCG), with highly variable efficacy rates ranging from 0 to 70% (Abubakar et al. 2013; Colditz et al. 1994). A number of factors contribute to BCG vaccine effectiveness, including variations in the vaccine strain or preparation, environment, previous exposure to *M. tuberculosis*, and host genetic background. Recent efforts to explore the role of host genetics on vaccine effectiveness have utilized mouse models. Using animals from the collaborative cross, described in detail below, Smith et al. reported BCG efficacy is controlled independently of susceptibility to TB in naïve animals (Smith et al. 2016). While no genes or loci were identified that directly contribute to vaccine efficacy, the study suggested that strain differences in immune responses may be responsible. These data suggest optimizing vaccines in non-TB responsive strains may be a more effective strategy than using only one standard mouse strain.

### *Streptococcus pneumoniae*

Infection with *Streptococcus pneumoniae* is the most common identifiable cause of pneumonia (Poll and Opal 2009) and infection triggers an inflammatory cascade response involving pattern recognition receptors, activation of NF-κB, and production of cytokines and chemokines (Quinton et al. 2007). Mortality remains high [between 5 and 35% (Brandenburg et al. 2000)] despite treatment with antibiotics, suggesting underlying host factors including genetic background may contribute to adverse outcomes or susceptibility. The contribution of host genetic background is also supported by differential susceptibility to *Streptococcus pneumoniae*-induced disease in inbred mouse strains (Gingles et al. 2001).

Several SNPs in innate and adaptive immunity genes have been identified in humans that either confer protection against or susceptibility to pneumococcal disease. A protective variant has been found in toll-interleukin 1 receptor domain-containing adaptor protein (*TIRAP*), which is responsible for signaling after toll-like receptor activation (Khor et al. 2007; Kumpf and Schumann 2010). The most well-characterized variant, *TIRAP* S180L, causes altered NF-κB signaling and protection against excessive inflammation induced by infection. In addition to protection against pneumococcal disease, the *TIRAP* S180L variant has been associated with protection against other bacterial diseases, e.g., TB and sepsis (Khor et al. 2007; Hamann et al. 2009). Additional protective SNPs in members of the NF-κB pathway, including *NFKBIA* and *NFKBIE*, were identified in a population from the United Kingdom (Chapman et al. 2007). SNPs in other immunity genes have been associated with susceptibility to *S. pneumoniae* infection including interleukin-17A (*IL17A*) and mannose-binding lectin (*MBL*) (Vuononvirta et al. 2015; Ozkan et al. 2012; Roy et al. 2002). As with many disease-associated polymorphisms, several other studies have found no significant association between bacterial diseases and SNPs in *TIRAP* or *MBL*, and bacterial diseases (Hawn et al. 2006; Miao et al. 2011; Endeman et al. 2008; Lundbo and Benfield 2017). Discrepancies among studies can arise from genotyping errors, differences in genotype frequencies between different populations, phenotypes chosen for analysis, population size, and failure to consider SNPs in linkage disequilibrium with the candidate SNP. Efforts to combine data from multiple studies into shared consortia, as with TB, may alleviate some of the caveats from single studies. An additional emerging tool to identify potentially causative genes is transcriptome-wide association studies (TWAS). This approach integrates gene expression data with large-scale GWAS to identify *cis*-regulated genes associated with complex traits (Gusev et al. 2016). Combined with GWAS consortia, TWAS may offer additional insights into host genetic contribution to infectious disease.

## Host genetic susceptibility to viral infection

### Respiratory syncytial virus (RSV)

RSV is the leading cause of lower respiratory tract hospitalizations in young children globally, and nearly everyone is infected with RSV by age 2 (Lozano et al. 2012). Estimated global ‘all ages mortality’ due to RSV was 235,000 in 2010 (Lozano et al. 2012). For most infants, symptoms resemble the common cold, however, others develop severe RSV disease requiring hospitalization and a subset of those infants require intensive care and disease may be so severe it results in death. In infants aged 0–17 days, RSV accounted for > 65,000 deaths, and in infants 28–364 days old, RSV accounted for > 136,000 deaths (Lozano et al. 2012). Importantly, no approved vaccine has been developed to protect against RSV infection. It is therefore critical that a more complete understanding of factors that contribute to severe RSV disease susceptibility is identified, including genetic background, so appropriate prevention and disease treatment strategies may be developed to reduce global burden of morbidity and mortality.

Known risk factors for severe RSV disease include prematurity, chronic lung disease, congenital heart disease, lack of breastfeeding, male sex, and immunodeficiency (Shi et al. 2015; Zhang et al. 2014). Despite these known risk factors, most infants that present with severe RSV bronchiolitis were previously healthy. Children that survive hospitalization for severe RSV disease are at increased risk of developing childhood wheezing and allergic asthma in adolescence (Sigurs et al. 2005). The wide variation in response to RSV infection suggests disease and susceptibility may arise due to differences in host factors (Amanatidou et al. 2009; Miyairi and DeVincenzo 2008).

Only one human GWA study has been done for RSV bronchiolitis that found several suggestive associations, one of which was also an expression QTL in *KCND3* (potassium voltage-gated channel subfamily D member 3) (Pasanen et al. 2017). Therefore, many of the investigations of genetic determinants for susceptibility to RSV infection and disease severity have been done using animal or cell models. In human populations, association studies have been performed with SNPs in biologically plausible candidate genes. As with other infectious diseases, most candidate gene studies for RSV infection and disease have focused on genes involved in immune pathways such as Toll-like receptor 4 (*TLR4*), cluster of differentiation 14 (*CD14*), surfactant proteins, cytokines, and chemokines (Awomoyi et al. 2007; Gentile et al. 2003; Paulus et al. 2007; Puthothu et al. 2006a, b, 2007a, b). The most comprehensive candidate gene study to date analyzed SNPs in 220 candidate immunity genes in a cohort of 470 children hospitalized in the Netherlands for severe RSV bronchiolitis (Janssen et al. 2007). The study found significant associations in several innate immunity genes, although only SNPs in the vitamin D receptor (*VDR*) were confirmed in a separate population from South Africa (Kresfelder et al. 2011).

Polymorphisms in *TLR4* have been repeatedly investigated, but associations with severe RSV infection/disease are conflicting (Paulus et al. 2007; Puthothu et al. 2006a, b; Goutak et al. 2014; Zhu et al. 2007). A recent meta-analysis of *TLR4* SNPs found no association with severity of RSV disease (Zhou et al. 2016). However, the meta-analysis did not include a study that examined *TLR4* genotypes with environmental exposures to lipopolysaccharide (LPS) that did show significant associations with RSV bronchiolitis in two independent populations (Caballero et al. 2015). Caballero et al. (2015) found that infants with the *TLR4* Asp299Gly heterozygous genotype from urban homes with low levels of LPS were at increased risk of developing severe RSV bronchiolitis. This contrasted with infants with the same Asp299Gly heterozygous genotype from rural homes with high levels of LPS who were more likely to have mild RSV disease. These results suggest that environmental factors via interaction with the *TLR4* genotype may modify the immune system to alter the way in which it responds to subsequent pathogens or insults. Studies like this also highlight the need to consider how the environment and the genome interact in determining an individual’s risk of disease susceptibility or severity. Perhaps if earlier studies of *TLR4* polymorphisms had also accounted for the home environments, more significant associations would have been found with severe RSV disease.

Translational studies by our group have identified additional candidate genes for RSV disease severity. Using an in vitro screen of RSV-infected human, genetically well-characterized lymphoblastoid cell lines from the HapMap project, we identified a candidate gene involved in inflammation, myxovirus (influenza virus) resistance 1 (*MX1*), with a functional loss-of-function SNP that associated with differential basal expression of *MX1* by the lymphoblastoid cell lines in vitro (Ciencewicki et al. 2014). Interestingly, the *MX1* SNP was also significantly associated with RSV disease severity in two populations of RSV-infected infants from Argentina (Ciencewicki et al. 2014). That is, infants with severe RSV disease were significantly more like to carry the *MX1* SNP than infants with mild RSV disease. This human in vitro/population study illustrates the translational value of using well-characterized genetic cell models to inform genetic contributions to human disease.

A few inbred mouse models of RSV infection and disease have also been developed to determine the genetic basis of susceptibility. The usefulness of inbred strains of animals for identification of genes that contribute to complex traits, and their application as translational tools to understand homologous traits in human populations, has been discussed elsewhere in this special edition of Mammalian Genome (e.g., Vellers et al). Using a panel of inbred strains of mice and analyses of *F*_1_, *F*_2_, and back-cross progeny from RSV susceptible Balb/c and resistant C3H/HeN strains of mice, Tregoning et al. (2010) found the MHC region was critical to the inflammatory sequelae following primary neonatal RSV infection. Stark et al. (2010) performed genome-wide linkage analyses of RSV infectivity in back-cross and *F*_2_ populations derived from RSV-resistant C57BL/6J and -susceptible AKR/J mice and identified a QTL on chromosome 6 that associated with increased susceptibility to infection. We performed a genome-wide association study using 30 inbred mouse strains and identified *Marco* as another immunity candidate gene (High et al. 2016), which as mentioned above has also been associated with tuberculosis susceptibility. Targeted deletion of *Marco* enhanced the susceptibility to RSV-induced lung inflammation and injury, consistent with the mouse GWAS findings. To investigate whether this gene is also important in humans, we identified and characterized a loss-of-function SNP in the *MARCO* promotor that significantly associated with RSV disease severity in two populations of RSV-infected infants (High et al. 2016). These studies highlight the utility of in vitro and in vivo models to identify candidate susceptibility genes that translate to human populations.

### Influenza virus

General risk factors for susceptibility to severe disease after seasonal influenza infection include extremes of age and a compromised immune system. Human genetic variation is thought to be at least partially responsible for the yearly influenza death rate that occurs despite widespread annual vaccination efforts. A study based in Utah found significant evidence for a heritable predisposition to death due to influenza infection (Albright et al. 2008).

Typical risk factors do not often predict influenza severity during a pandemic since more severe disease occurs in otherwise healthy adults (Girard et al. 2010). The most recent influenza pandemic in 2009 (strain pH1N1) has been extensively studied to investigate the contribution of genetic variation in humans on disease severity (Maestri et al. 2015; Zhang et al. 2013; Zuniga et al. 2012). Several SNPs, particularly in immunity genes, have been correlated with severe disease in a variety of populations. For example, in a Canadian population, a polymorphism in the *CCR5* gene (*CCR5*Δ32) was overrepresented in Caucasians with severe influenza infection (Keynan et al. 2010). In a Chinese population, the complement regulatory immunity gene *CD55*, was found to associate with pH1N1 disease severity, where an allele-specific effect of promotor variant rs2564978 (genotype T/T exhibited significantly lower transcriptional activity than that with genotype C/C) and a functional indel variation of rs3841376 were found as genetic markers for severe pH1N1 disease (Zhou et al. 2012). Multiple other studies have identified a SNP-mediated splice variant in interferon-induced transmembrane protein 3 (*IFITM3*), a gene important in influenza virus replication and associated with influenza susceptibility and disease severity in Caucasian and Asian populations (Zhang et al. 2013; Everitt et al. 2012, 80; Yang et al. 2015; Allen et al. 2017).

As with other infectious diseases, mouse models have been used to discover candidate genes for influenza pathogenesis. Crosses between C57BL/6J, a relatively influenza resistant strain, and DBA/2J, a highly susceptible strain, have revealed multiple QTLs with potential candidate genes involved in influenza responsiveness (Boon et al. 2009; Nedelko et al. 2012). Many of the potential candidate genes are part of immune pathways. Similar to RSV, SNPs in *Mx1* are associated with influenza pathogenesis, including a novel *Mx1* allele identified using collaborative cross mice (Ferris et al. 2013a, b; Staeheli et al. 1988). Taking advantage of genetic diversity in mouse models should aid in translation to human populations and is discussed in more detail below.

Not only does host genetic background contribute to susceptibility to influenza infection but it also contributes to vaccine responsiveness. A few polymorphisms in immunity genes have been significantly associated with responsiveness to vaccination against influenza, particularly in HLA class II alleles. Individuals with the HLA-DRB1*07 allele produce lower antibody titers following administration of the influenza vaccine (Gelder et al. 2002). This same study also found a lower frequency of HLA-DBQ*0603-9/14 in vaccine non-responders. In an elderly cohort, the HLA-DRB1*04:01 and HLA-DPB1*04:01 variants were found at higher frequencies in vaccine responders compared to non-responders (Moss et al. 2013). SNPs in several cytokine and cytokine receptor genes (*IL6, IL8, IL12A, IL12B, IFNG, IL1R, IL2RG, IL4R, IL10RB, IL12RB, IFNAR2, TNFRS F1A*) are also significantly associated with influenza H1N1 antibody titers (Poland et al. 2008).

Sex differences in response to influenza vaccination have also been reported, further highlighting that host genetic background is an important determinant of responsiveness. A few studies have reported females have greater antibody responses to influenza vaccination than males (Engler et al. 2008; Falsey et al. 2009; Giefing-Kröll et al. 2015). However, it is unclear why females tend to have a more effective immune response after vaccination. The primary hypothesis is that differences in sex steroid hormones, which are known to have differing effects on the immune system, contribute to the sexual dimorphism. For example, testosterone is thought to have an immunosuppressive role in response to influenza vaccination via repression of immune activation transcription factors and higher expression of lipid metabolism genes (Furman et al. 2014). While sex is an important determinant of influenza vaccine efficacy, it is unclear if there are sex differences in susceptibility to influenza infection, with the exception of pregnancy as a known risk factor for enhanced risk of severe influenza (Gabriel and Arck 2014).

### Summary and future directions

This brief review of studies in human populations and inbred animal models has illustrated the importance of genetic background as a critical component to differential susceptibility to respiratory infectious disease and, potentially, to the development of vaccines to prevent these diseases. While the use of association study approaches to identify the importance of gene candidates in human populations has strengths, there are also weaknesses. For example, as evident from the human studies mentioned in the previous sections, one of the prevailing limitations is the inconsistencies of associated gene candidates. With human-based studies, especially those that are retrospective, there is an inability to control for all confounding variables such as diet, environmental exposures, and activity. Furthermore, the studies described in this review directly impact the lung, and thus presenting another limitation in that human studies only can examine certain aspects of the lung (e.g., specific cell types), and not the contribution(s) of different components of the lung (e.g., different lobes, bronchioles, alveoli). Therefore, such limitations in human studies could underlie the inconsistent findings across studies describing inter-individual variability to infectious respiratory illnesses. Other caveats with genetic association studies are the lack of replication within and between studies, genotyping errors, and population stratification. To better understand potential weaknesses in genetic association studies, the STREGA (Strengthening the reporting of genetic association studies) statement was created (Little et al. 2009). The STREGA recommendations do not prescribe study design, but rather recommend how to enhance reporting transparency regardless of study design or analyses (Little et al. 2009). Moreover, genetic association studies by design usually investigate gene candidates that have already been found to have biological plausibility and therefore novel gene candidates will likely not be identified. To avoid this limitation, GWAS approaches have been employed to identify novel genes that contribute to the disease or phenotype without a priori hypotheses. However, GWAS approaches also have their own caveats which have been reviewed elsewhere (e.g., Ober 2016). While GWAS have identified many genes that have been replicated independently, most contributions to disease risk are small (i.e., the variants account for a small fraction of the heritability of complex diseases). Future GWAS designs should consider gene–environment interactions, as many diseases also have an important environmental component that is often difficult to quantify and incorporate into genetic investigations of disease variants. Systems genetics approaches have also been employed to complement GWAS to better understand the genetic and genomic contributions to disease heritability (Bjorkegren et al. 2015).

Given study limitations with human populations, mammalian animal models remain necessary to control for external confounding variables and to allow access to the entirety of the lung (Fig. 1). While various inbred strains and genetics models have been used to identify genetic contributors to infectious respiratory disease susceptibility, such models present some limitations regarding translation of findings to humans. Inbred strains are useful in identifying some causal genetic factors but most investigations do not fully mimic the heterogeneity found in humans. Relatively new mouse models, known as the Collaborative Cross (CC) and Diversity Outbred (DO) models, offer promise to more closely mimic the genetic variability in humans and that have a controlled and defined genetic background (Roberts et al. 2007). Briefly, the CC mice are a large panel of multi-parent recombinant inbred strains derived from eight founder strains representing the three major mouse subspecies (A/J, C57Bl/6J, 129Sv/ImJ, NOD/LtJ, NZO/H1J, CAST/EiJ, PWK/PhJ, and WSB/EiJ) and that capture approximately 90% of the known genetic variation in laboratory mice with the captured variation being randomly distributed across the genome (Roberts et al. 2007; Churchill et al. 2004; Consortium 2012). The DO mice are derived from the same eight founder strains of the CC mice; however, the generation of DO mice begins with a randomized and strict breeding scheme of the CC mice so that each DO mouse is genetically unique (Schmidt 2015; Churchill et al. 2012). The CC mice have already been used in studies investigating infectious diseases including influenza (Bottomly et al. 2012; Ferris et al. 2013) and Ebola (Rasmussen et al. 2014), and the DO mouse model in TB (Niazi et al. 2015) and drug toxicity-related studies (Harrill 2016; Church et al. 2015). Interestingly, the drug toxicity studies utilizing the DO model have been able to identify phenotypic markers that are unidentifiable in the standard inbred mouse models (Harrill 2016).

Taken together, we suggest that future work investigating the relationship between respiratory infectious diseases and genetics consider employing animal models such as the CC and/or DO mouse models to unravel the genetic architecture of complex traits for translation into human models and development of vaccines. In addition, future GWAS designs should consider incorporating gene–environment and host–pathogen interactions to address these other factors that contribute to disease susceptibility.

## References

[CR1] Abubakar I, Pimpin L, Ariti C, Beynon R, Mangtani P, Sterne JA et al (2013) Systematic review and meta-analysis of the current evidence on the duration of protection by bacillus Calmette-Guerin vaccination against tuberculosis. Health Technol Assess 17(37):1–372, v–vi 10.3310/hta17370 PMC4781620 24021245

[CR2] Albright FS, Orlando P, Pavia AT, Jackson GG, Albright LAC (2008). Evidence for a heritable predisposition to death due to influenza. J Infect Dis.

[CR3] Allen EK, Randolph AG, Bhangale T, Dogra P, Ohlson M, Oshansky CM (2017). SNP-mediated disruption of CTCF binding at the IFITM3 promoter is associated with risk of severe influenza in humans. Nat Med.

[CR4] Amanatidou V, Apostolakis S, Spandidos DA (2009). Genetic diversity of the host and severe respiratory syncytial virus-induced lower respiratory tract infection. Pediatr Infect Dis J.

[CR5] Apt AS, Logunova NN, Kondratieva TK (2017). Host genetics in susceptibility to and severity of mycobacterial diseases. Tuberculosis.

[CR6] Awomoyi AA, Rallabhandi P, Pollin TI, Lorenz E, Sztein MB, Boukhvalova MS (2007). Association of TLR4 polymorphisms with symptomatic respiratory syncytial virus infection in high-risk infants and young children. J Immunol.

[CR7] Bellamy R, Ruwende C, Corrah T, McAdam KP, Whittle HC, Hill AV (1998). Variations in the NRAMP1 gene and susceptibility to tuberculosis in West Africans. N Engl J Med.

[CR8] Bjorkegren JL, Kovacic JC, Dudley JT, Schadt EE (2015). Genome-wide significant loci: how important are they? Systems genetics to understand heritability of coronary artery disease and other common complex disorders. J Am Coll Cardiol.

[CR9] Boon AC, deBeauchamp J, Hollmann A, Luke J, Kotb M, Rowe S (2009). Host genetic variation affects resistance to infection with a highly pathogenic H5N1 influenza A virus in mice. J Virol.

[CR10] Bottomly D, Ferris MT, Aicher LD, Rosenzweig E, Whitmore A, Aylor DL (2012). Expression quantitative trait Loci for extreme host response to influenza a in pre-collaborative cross mice. G3 Genes Genomes Genet.

[CR11] Brandenburg JA, Marrie TJ, Coley CM, Singer DE, Obrosky DS, Kapoor WN (2000). Clinical presentation, processes and outcomes of care for patients with pneumococcal pneumonia. J Gen Intern Med.

[CR12] Bustamante J, Boisson-Dupuis S, Abel L, Casanova JL (2014). Mendelian susceptibility to mycobacterial disease: genetic, immunological, and clinical features of inborn errors of IFN-gamma immunity. Semin Immunol.

[CR13] Caballero MT, Serra ME, Acosta PL, Marzec J, Gibbons L, Salim M (2015). TLR4 genotype and environmental LPS mediate RSV bronchiolitis through Th2 polarization. J Clin Invest.

[CR14] Caws M, Thwaites G, Dunstan S, Hawn TR, Lan NT, Thuong NT (2008). The influence of host and bacterial genotype on the development of disseminated disease with *Mycobacterium tuberculosis*. PLoS Pathog.

[CR15] Chapman SJ, Khor CC, Vannberg FO, Frodsham A, Walley A, Maskell NA (2007). IκB genetic polymorphisms and invasive pneumococcal disease. Am J Respir Crit Care Med.

[CR16] Church RJ, Gatti DM, Urban TJ, Long N, Yang X, Shi Q (2015). Sensitivity to hepatotoxicity due to epigallocatechin gallate is affected by genetic background in diversity outbred mice. Food Chem Toxicol.

[CR17] Churchill GA, Airey DC, Allayee H, Angel JM, Attie AD, Beatty J (2004). The Collaborative Cross, a community resource for the genetic analysis of complex traits. Nat Genet.

[CR18] Churchill GA, Gatti DM, Munger SC, Svenson KL (2012). The diversity outbred mouse population. Mamm Genome.

[CR19] Ciencewicki JM, Wang X, Marzec J, Serra ME, Bell DA, Polack FP (2014). A genetic model of differential susceptibility to human respiratory syncytial virus (RSV) infection. FASEB J.

[CR20] Colditz GA, Brewer TF, Berkey CS, Wilson ME, Burdick E, Fineberg HV (1994). Efficacy of BCG vaccine in the prevention of tuberculosis. Meta-analysis of the published literature. JAMA.

[CR21] GBD 2015 LRI Collaborators (2017). Estimates of the global, regional, and national morbidity, mortality, and aetiologies of lower respiratory tract infections in 195 countries: a systematic analysis for the Global Burden of Disease Study 2015. Lancet Infect Dis.

[CR22] Comstock GW (1978). Tuberculosis in twins: a re-analysis of the Prophit survey 1–3. Am Rev Respir Dis.

[CR23] Consortium CC (2012). The genome architecture of the Collaborative Cross mouse genetic reference population. Genetics.

[CR24] Davila S, Hibberd ML, Dass RH, Wong HE, Sahiratmadja E, Bonnard C (2008). Genetic association and expression studies indicate a role of toll-like receptor 8 in pulmonary tuberculosis. PLoS Genet.

[CR25] Endeman H, Herpers BL, de Jong BA, Voorn GP, Grutters JC, van Velzen-Blad H (2008). Mannose-binding lectin genotypes in susceptibility to community-acquired pneumonia. CHEST J.

[CR26] Engler RJ, Nelson MR, Klote MM, VanRaden MJ, Huang C-Y, Cox NJ (2008). Half-vs full-dose trivalent inactivated influenza vaccine (2004–2005): age, dose, and sex effects on immune responses. Arch Intern Med.

[CR27] Everitt AR, Clare S, Pertel T, John SP, Wash RS, Smith SE (2012). IFITM3 restricts the morbidity and mortality associated with influenza. Nature.

[CR28] Falsey AR, Treanor JJ, Tornieporth N, Capellan J, Gorse GJ (2009). Randomized, double-blind controlled phase 3 trial comparing the immunogenicity of high-dose and standard-dose influenza vaccine in adults 65 years of age and older. J Infect Dis.

[CR29] Ferris MT, Aylor DL, Bottomly D, Whitmore AC, Aicher LD, Bell TA (2013). Modeling host genetic regulation of influenza pathogenesis in the collaborative cross. PLoS Pathog.

[CR30] Furman D, Hejblum BP, Simon N, Jojic V, Dekker CL, Thiébaut R (2014). Systems analysis of sex differences reveals an immunosuppressive role for testosterone in the response to influenza vaccination. Proc Natl Acad Sci USA.

[CR31] Gabriel G, Arck PC (2014). Sex, immunity and influenza. J Infect Dis.

[CR32] Gelder CM, Lambkin R, Hart KW, Fleming D, Williams OM, Bunce M (2002). Associations between human leukocyte antigens and nonresponsiveness to influenza vaccine. J Infect Dis.

[CR33] Gentile DA, Doyle WJ, Zeevi A, Howe-Adams J, Kapadia S, Trecki J (2003). Cytokine gene polymorphisms moderate illness severity in infants with respiratory syncytial virus infection. Hum Immunol.

[CR34] Giefing-Kröll C, Berger P, Lepperdinger G, Grubeck-Loebenstein B (2015). How sex and age affect immune responses, susceptibility to infections, and response to vaccination. Aging Cell.

[CR35] Gingles NA, Alexander JE, Kadioglu A, Andrew PW, Kerr A, Mitchell TJ (2001). Role of genetic resistance in invasive pneumococcal infection: identification and study of susceptibility and resistance in inbred mouse strains. Infect Immun.

[CR36] Girard MP, Tam JS, Assossou OM, Kieny MP (2010). The 2009 A (H1N1) influenza virus pandemic: a review. Vaccine.

[CR37] Goutak M, Haidopoulou K, Pappa S, Tsakipjdis P, Frydas E, Eboriadou M (2014). The role of TLR4 and CD14 polymorphisms in the pathogenesis of respiratory syncytial virus bronchiolitis in greek infants. Int J Immunopathol Pharmacol.

[CR38] Gusev A, Ko A, Shi H, Bhatia G, Chung W, Penninx BW (2016). Integrative approaches for large-scale transcriptome-wide association studies. Nat Genet.

[CR39] Hamann L, Kumpf O, Schuring RP, Alpsoy E, Bedu-Addo G, Bienzle U (2009). Low frequency of the TIRAP S180L polymorphism in Africa, and its potential role in malaria, sepsis, and leprosy. BMC Med Genet.

[CR40] Harrill AH, Will Y, McDuffie JE, Olaharski AJ, Jeffy BD (2016). Mouse population-based toxicology for personalized medicine and improved safety prediction. Drug discovery toxicology: from target assessment to translational biomarkers.

[CR41] Hawn TR, Dunstan SJ, Thwaites GE, Simmons CP, Thuong NT, Lan NTN (2006). A polymorphism in Toll-interleukin 1 receptor domain containing adaptor protein is associated with susceptibility to meningeal tuberculosis. J Infect Dis.

[CR42] High M, Cho H-Y, Marzec J, Wiltshire T, Verhein KC, Caballero MT (2016). Determinants of host susceptibility to murine respiratory syncytial virus (RSV) disease identify a role for the innate immunity scavenger receptor MARCO gene in human infants. EBioMedicine.

[CR43] Huang L, Liu C, Liao G, Yang X, Tang X, Chen J (2015). Vitamin D receptor gene foki polymorphism contributes to increasing the risk of tuberculosis: an update meta-analysis. Medicine.

[CR44] Janssen R, Bont L, Siezen CL, Hodemaekers HM, Ermers MJ, Doornbos G (2007). Genetic susceptibility to respiratory syncytial virus bronchiolitis is predominantly associated with innate immune genes. J Infect Dis.

[CR45] Keynan Y, Card CM, Ball BT, Li Y, Plummer FA, Fowke KR (2010). Cellular immune responses to recurring influenza strains have limited boosting ability and limited cross-reactivity to other strains. Clin Microbiol Infect.

[CR46] Khor CC, Chapman SJ, Vannberg FO, Dunne A, Murphy C, Ling EY (2007). A functional variant in TIRAP, also known as MAL, and protection against invasive pneumococcal disease, bacteraemia, malaria and tuberculosis. Nat Genet.

[CR47] Kinnear C, Hoal EG, Schurz H, van Helden PD, Möller M (2017). The role of human host genetics in Tuberculosis resistance. Expert Rev Respir Med.

[CR48] Kramnik I, Beamer G (2016). Mouse models of human TB pathology: roles in the analysis of necrosis and the development of host-directed therapies. Semin Immunopathol.

[CR49] Kramnik I, Dietrich WF, Demant P, Bloom BR (2000). Genetic control of resistance to experimental infection with virulent *Mycobacterium tuberculosis*. Proc Natl Acad Sci USA.

[CR50] Kresfelder T, Janssen R, Bont L, Venter M (2011). Confirmation of an association between single nucleotide polymorphisms in the VDR gene with respiratory syncytial virus related disease in South African children. J Med Virol.

[CR51] Kumpf O, Schumann RR (2010). Genetic variation in innate immunity pathways and their potential contribution to the SIRS/CARS debate: evidence from human studies and animal models. J Innate Immun.

[CR52] Lao W, Kang H, Jin G, Chen L, Chu Y, Sun J (2017). Evaluation of the relationship between MARCO and CD36 single-nucleotide polymorphisms and susceptibility to pulmonary tuberculosis in a Chinese Han population. BMC Infect Dis.

[CR53] Little J, Higgins JP, Ioannidis JP, Moher D, Gagnon F, von Elm E (2009). STrengthening the REporting of Genetic Association Studies (STREGA): an extension of the STROBE statement. PLoS Med.

[CR54] Logunova N, Korotetskaya M, Polshakov V, Apt A (2015). The QTL within the H2 complex involved in the control of tuberculosis infection in mice is the classical class II H2-Ab1 gene. PLoS Genet.

[CR55] Lozano R, Naghavi M, Foreman K, Lim S, Shibuya K, Aboyans V (2012). Global and regional mortality from 235 causes of death for 20 age groups in 1990 and 2010: a systematic analysis for the Global Burden of Disease Study 2010. Lancet.

[CR56] Lundbo LF, Benfield T (2017). Risk factors for community-acquired bacterial meningitis. Infect Dis.

[CR57] Maestri A, Sortica VA, Tovo-Rodrigues L, Santos MC, Barbagelata L, Moraes MR (2015). Siaα2-3Galβ1-receptor genetic variants are associated with influenza A (H1N1) pdm09 severity. PLoS ONE.

[CR58] Medina E, North RJ (1998). Resistance ranking of some common inbred mouse strains to *Mycobacterium tuberculosis* and relationship to major histocompatibility complex haplotype and Nramp1 genotype. Immunology.

[CR59] Meilang Q, Zhang Y, Zhang J, Zhao Y, Tian C, Huang J (2012). Polymorphisms in the SLC11A1 gene and tuberculosis risk: a meta-analysis update. Int J Tuberc Lung Dis.

[CR60] Miao R, Li J, Sun Z, Xu F, Shen H (2011). Meta-analysis on the association of TIRAP S180L variant and tuberculosis susceptibility. Tuberculosis.

[CR61] Miyairi I, DeVincenzo JP (2008). Human genetic factors and respiratory syncytial virus disease severity. Clin Microbiol Rev.

[CR62] Moss AJ, Gaughran FP, Karasu A, Gilbert AS, Mann AJ, Gelder CM (2013). Correlation between human leukocyte antigen class II alleles and HAI titers detected post-influenza vaccination. PLoS ONE.

[CR63] Naranbhai V (2016). The role of host genetics (and genomics) in tuberculosis. Microbiol Spectr.

[CR64] Nedelko T, Kollmus H, Klawonn F, Spijker S, Lu L, Hessman M (2012). Distinct gene loci control the host response to influenza H1N1 virus infection in a time-dependent manner. BMC Genom.

[CR65] Niazi MK, Dhulekar N, Schmidt D, Major S, Cooper R, Abeijon C (2015). Lung necrosis and neutrophils reflect common pathways of susceptibility to *Mycobacterium tuberculosis* in genetically diverse, immune-competent mice. Dis Model Mech.

[CR66] Ober C (2016). Asthma genetics in the post-GWAS era. Ann Am Thorac Soc.

[CR67] Omae Y, Toyo-Oka L, Yanai H, Nedsuwan S, Wattanapokayakit S, Satproedprai N et al (2017) Pathogen lineage-based genome-wide association study identified CD53 as susceptible locus in tuberculosis. J Hum Genet 10.1038/jhg.2017.82 PMC5709719 28878339

[CR68] Orme IM (2003). The mouse as a useful model of tuberculosis. Tuberculosis.

[CR69] Ozkan H, Koksal N, Cetinkaya M, Kilic S, Celebi S, Oral B (2012). Serum mannose-binding lectin (MBL) gene polymorphism and low MBL levels are associated with neonatal sepsis and pneumonia. J Perinatol.

[CR70] Pan H, Yan BS, Rojas M, Shebzukhov YV, Zhou H, Kobzik L (2005). Ipr1 gene mediates innate immunity to tuberculosis. Nature.

[CR71] Pasanen A, Karjalainen MK, Bont L, Piippo-Savolainen E, Ruotsalainen M, Goksor E (2017). Genome-wide association study of polymorphisms predisposing to bronchiolitis. Sci Rep.

[CR72] Paulus SC, Hirschfeld AF, Victor RE, Brunstein J, Thomas E, Turvey SE (2007). Common human Toll-like receptor 4 polymorphisms—role in susceptibility to respiratory syncytial virus infection and functional immunological relevance. Clin Immunol.

[CR73] Poland GA, Ovsyannikova IG, Jacobson RM (2008). Immunogenetics of seasonal influenza vaccine response. Vaccine.

[CR74] Puthothu B, Krueger M, Heinze J, Forster J, Heinzmann A (2006). Impact of IL8 and IL8-receptor alpha polymorphisms on the genetics of bronchial asthma and severe RSV infections. Clin Mol Allergy.

[CR75] Puthothu B, Krueger M, Heinze J, Forster J, Heinzmann A (2006). Haplotypes of surfactant protein C are associated with common paediatric lung diseases. Pediatr Allergy Immunol.

[CR76] Puthothu B, Forster J, Heinze J, Heinzmann A, Krueger M (2007). Surfactant protein B polymorphisms are associated with severe respiratory syncytial virus infection, but not with asthma. BMC Pulm Med.

[CR77] Puthothu B, Krueger M, Forster J, Heinze J, Weckmann M, Heinzmann A (2007). Interleukin (IL)-18 polymorphism 133C/G is associated with severe respiratory syncytial virus infection. Pediatr Infect Dis J.

[CR78] Quinton LJ, Jones MR, Simms BT, Kogan MS, Robson BE, Skerrett SJ (2007). Functions and regulation of NF-κB RelA during pneumococcal pneumonia. J Immunol.

[CR79] Rasmussen AL, Okumura A, Ferris MT, Green R, Feldmann F, Kelly SM (2014). Host genetic diversity enables Ebola hemorrhagic fever pathogenesis and resistance. Science.

[CR80] Roberts A, De Villena FP-M, Wang W, McMillan L, Threadgill DW (2007). The polymorphism architecture of mouse genetic resources elucidated using genome-wide resequencing data: implications for QTL discovery and systems genetics. Mamm Genome.

[CR81] Roy S, Knox K, Segal S, Griffiths D, Moore CE, Welsh KI (2002). MBL genotype and risk of invasive pneumococcal disease: a case-control study. Lancet.

[CR82] Salie M, van der Merwe L, Moller M, Daya M, van der Spuy GD, van Helden PD (2014). Associations between human leukocyte antigen class I variants and the *Mycobacterium tuberculosis* subtypes causing disease. J Infect Dis.

[CR83] Salie M, Daya M, Lucas LA, Warren RM, van der Spuy GD, van Helden PD (2015). Association of toll-like receptors with susceptibility to tuberculosis suggests sex-specific effects of TLR8 polymorphisms. Infect Genet Evol.

[CR84] Schmidt CW (2015). Diversity outbred: a new generation of mouse model. Environ Health Perspect.

[CR85] Shi T, Balsells E, Wastnedge E, Singleton R, Rasmussen ZA, Zar HJ (2015). Risk factors for respiratory syncytial virus associated with acute lower respiratory infection in children under five years: systematic review and meta-analysis. J Glob Health.

[CR86] Sigurs N, Gustafsson PM, Bjarnason R, Lundberg F, Schmidt S, Sigurbergsson F (2005). Severe respiratory syncytial virus bronchiolitis in infancy and asthma and allergy at age 13. Am J Respir Crit Care Med.

[CR87] Smith CM, Proulx MK, Olive AJ, Laddy D, Mishra BB, Moss C (2016). Tuberculosis susceptibility and vaccine protection are independently controlled by host genotype. MBio.

[CR88] Staeheli P, Grob R, Meier E, Sutcliffe JG, Haller O (1988). Influenza virus-susceptible mice carry Mx genes with a large deletion or a nonsense mutation. Mol Cell Biol.

[CR89] Stark JM, Barmada MM, Winterberg AV, Majumber N, Gibbons WJ, Stark MA (2010). Genomewide association analysis of respiratory syncytial virus infection in mice. J Virol.

[CR90] Stead WW, Senner JW, Reddick WT, Lofgren JP (1990). Racial differences in susceptibility to infection by *Mycobacterium tuberculosis*. N Engl J Med.

[CR91] Sudre P, Ten Dam G, Kochi A (1992). Tuberculosis: a global overview of the situation today. Bull World Health Organ.

[CR92] Thye T, Niemann S, Walter K, Homolka S, Intemann CD, Chinbuah MA (2011). Variant G57E of mannose binding lectin associated with protection against tuberculosis caused by Mycobacterium africanum but not by *M. tuberculosis*. PLoS ONE.

[CR93] Tong X, Chen L, Liu S, Yan Z, Peng S, Zhang Y (2015). Polymorphisms in HLA-DRB1 gene and the risk of tuberculosis: a meta-analysis of 31 studies. Lung.

[CR94] Tregoning JS, Yamaguchi Y, Wang B, Mihm D, Harker JA, Bushell ES (2010). Genetic susceptibility to the delayed sequelae of neonatal respiratory syncytial virus infection is MHC dependent. J Immunol.

[CR95] van der Poll T, Opal SM (2009). Pathogenesis, treatment, and prevention of pneumococcal pneumonia. Lancet.

[CR96] Vuononvirta J, Peltola V, Ilonen J, Mertsola J, He Q (2015). The gene polymorphism of IL-17 G-152A is associated with increased colonization of *Streptococcus pneumoniae* in young Finnish children. Pediatr Infect Dis J.

[CR97] World Health Organization (2013). Global tuberculosis report 2013.

[CR98] Xu C, Tang P, Ding C, Li C, Chen J, Xu Z (2015). Vitamin D receptor gene FOKI polymorphism contributes to increasing the risk of HIV-negative tuberculosis: evidence from a meta-analysis. PLoS ONE.

[CR99] Xuan Y, Wang L, Li W, Zi H, Guo Y, Yan W (2015). IFITM3 rs12252 T>C polymorphism is associated with the risk of severe influenza: a meta-analysis. Epidemiol Infect.

[CR100] Yang X, Tan B, Zhou X, Xue J, Zhang X, Wang P (2015). Interferon-inducible transmembrane protein 3 genetic variant rs12252 and influenza susceptibility and severity: a meta-analysis. PLoS ONE.

[CR101] Yang K, Hung J, Lin P (2016). HLA-DRB1* 16: 39, a novel HLA-DRB1* 16 variant, discovered in a Taiwanese bone marrow hematopoietic stem cell donor. Hla.

[CR102] Zhang Y-H, Zhao Y, Li N, Peng Y-C, Giannoulatou E, Jin R-H (2013). Interferon-induced transmembrane protein-3 genetic variant rs12252-C is associated with severe influenza in Chinese individuals. Nat Commun.

[CR103] Zhang XB, Liu LJ, Qian LL, Jiang GL, Wang CK, Jia P (2014). Clinical characteristics and risk factors of severe respiratory syncytial virus-associated acute lower respiratory tract infections in hospitalized infants. World J Pediatr.

[CR104] Zhou J, To KK-W, Dong H, Cheng Z-S, Lau CC-Y, Poon VK (2012). A functional variation in CD55 increases the severity of 2009 pandemic H1N1 influenza A virus infection. J Infect Dis.

[CR105] Zhou J, Zhang X, Liu S, Wang Z, Chen Q, Wu Y (2016). Genetic association of TLR4 Asp299Gly, TLR4 Thr399Ile, and CD14 C-159T polymorphisms with the risk of severe RSV infection: a meta-analysis. Influenza Other Respir Viruses.

[CR106] Zhu J, Martinez J, Huang X, Yang Y (2007). Innate immunity against vaccinia virus is mediated by TLR2 and requires TLR-independent production of IFN-β. Blood.

[CR107] Zuniga J, Buendia-Roldan I, Zhao Y, Jimenez L, Torres D, Romo J (2012). Genetic variants associated with severe pneumonia in A/H1N1 influenza infection. Eur Respir J.

